# An overview of self-supervised deep learning applications to molecular data

**DOI:** 10.1093/bib/bbag380

**Published:** 2026-07-17

**Authors:** Lluis Borràs Ferrís, Riccardo Fratti, Valerio Nucera, Valentin Oreiller, Barbara Di Camillo, Manfredo Atzori, Henning Müller

**Affiliations:** Institute of Informatics, University of Applied Sciences Western Switzerland (HES-SO), Technopôle 3, 3960 Sierre, Valais, Switzerland; Nuclear Medicine and Molecular Imaging Department, Lausanne University Hospital (CHUV), Rue du Bugnon 46, 1011 Lausanne, Vaud, Switzerland; Institute of Informatics, University of Applied Sciences Western Switzerland (HES-SO), Technopôle 3, 3960 Sierre, Valais, Switzerland; Institute of Informatics, University of Applied Sciences Western Switzerland (HES-SO), Technopôle 3, 3960 Sierre, Valais, Switzerland; Institute of Informatics, University of Applied Sciences Western Switzerland (HES-SO), Technopôle 3, 3960 Sierre, Valais, Switzerland; Information Engineering Department, University of Padua, Via Gradenigo 6/b, 35131 Padua, Italy; Institute of Informatics, University of Applied Sciences Western Switzerland (HES-SO), Technopôle 3, 3960 Sierre, Valais, Switzerland; Department of Neuroscience, University of Padua, Via Belzoni 160, 35121 Padua, Italy; Institute of Informatics, University of Applied Sciences Western Switzerland (HES-SO), Technopôle 3, 3960 Sierre, Valais, Switzerland; The Sense Innovation and Research Center, Avenue de Provence 82, 1007 Lausanne / Rue de l'Industrie 17, 1950 Sion, Switzerland

**Keywords:** self-supervised learning, unsupervised learning, molecular data, feature representation, foundation models, contrastive learning

## Abstract

The emergence of high-throughput sequencing technologies has generated unprecedented amounts of molecular data, posing significant challenges for analysis and interpretation. In the past 15 years, deep learning (DL) has revolutionized many data analysis fields. However, a key limitation of most DL approaches is their reliance on massive amounts of data that often require labeling. Self-supervised learning (SSL) uses large-scale unlabeled data to learn meaningful representations for specific tasks, allowing training of the models without labels. While SSL has primarily been applied to fields like natural language processing and medical image analysis, SSL can also be applied to molecular data to learn meaningful representations of molecular sequences that can be used for downstream tasks. Despite the growing interest in SSL applications using molecular data over the recent years, no comprehensive review has been published on this topic, so far, making it timely to address. This paper aims to provide researchers in DL and bioinformatics with a clear view of SSL omics applications to foster future work in the domain. This review examines the principles of SSL, such as foundation models, and it discusses the application of SSL to various omics data types, summarizes information from 17 studies, and categorizes applications by data type, detailing common tasks, model architectures, and repositories. Key applications such as DNABERT and Nucleotide Transformer are highlighted, demonstrating the contributions of SSL in understanding gene regulation. Future directions for SSL in omics are outlined, emphasizing the potential for integrating multi-omics data and developing more sophisticated pretext tasks.

## Introduction

 Genomics research is broadly defined as the study of an organism’s complete set of DNA. A major goal is to characterize all genes and functional elements in an organism’s genome, to understand how they are regulated, and how they are related with diseases. While modern high-throughput technologies, such as whole-genome sequencing (WGS), RNA sequencing (RNA-seq), and single-cell RNA sequencing (scRNA-seq), provide a comprehensive view of how genes and their regulation drive biological processes, they also pose a significant challenge for data analysis [1].

Deep learning (DL) enables computational models with many processing layers to capture and learn data representations [2]. DL techniques involve end-to-end training of models, allowing them to learn directly from raw data. Unlike traditional machine learning algorithms, DL models utilize thousands, millions, or even billions of parameters to automatically discover and characterize the data during training, allowing them to model complex and unknown relations among data. DL models were traditionally developed under a strongly supervised paradigm, requiring large amounts of high-quality labeled data [3, 4]. This approach proved successful in biology, most notably with models, such as AlphaFold [5, 6], DeepSEA [7], and Enformer [8]. However, supervised training relies heavily on expert-curated labels, which are costly, time-consuming, and often limited in biological settings. In parallel, the breakthrough of transformer-based self-supervised models, such as BERT [9] and GPT [10] in natural language processing (NLP), and MoCo [11] and SimCLR [12] in computer vision, profoundly reshaped the field of machine learning. These successes established a new paradigm: pretraining large foundation models (FMs) on unlabeled data using self-supervised learning (SSL), followed by task-specific fine-tuning.

SSL has the potential to open new avenues for discovering patterns and insights from the vast amounts of unlabeled data available in the omics domain, unlike supervised methods that require large amounts of labeled data that are often expensive to obtain in genomics [13].

However, despite the increasing interest in SSL applications using molecular sequence data in recent years, so far, no comprehensive review has been published. Given the rapid advances and potential impact of SSL in addressing outstanding challenges in omics, a thorough review is timely and essential to guide future research and applications in this evolving field.

This review aims to provide a clear view of the current state of SSL research using molecular data, with the goal of providing precise insights for scientists in DL and bioinformatics.

This paper presents a comprehensive survey of models pretrained using SSL and their applications using molecular data, focusing on the latest advancements and key contributions to the omics field. The applications of SSL in omics are categorized based on the type of data used, including WGS, RNA-seq, scRNA-seq, and protein sequences. The review is a resource for reproducibility, providing information on available code repositories in genetics for the SSL models and datasets, promoting further research and innovation in the field.

## Molecular data

Omics studies were revolutionized by the development of high-throughput sequencing technologies, generating petabytes of data, the majority of which are released in public repositories such as gene expression omnibus (GEO) and The sequence read archive (SRA) [14]. However, due to the data complexity and scale, extracting meaningful biological insights remains a significant challenge.

Molecular data includes various types such as genomic sequences (DNA), transcriptomic data including RNA sequences and gene expression levels (of bulk and single-cell transcriptomics), and proteomic data, comprising both protein sequences and expression profiles. High-throughput technologies, such as next-generation sequencing, have enabled the collection of these data at an unprecedented scale. The study of each modality presents unique challenges and opportunities for analysis using DL techniques. DL has already shown its applications using molecular data with the examples of AlphaFold [5, 6] for protein structure applications, DeepSEA [7] for a better understanding of the effect of noncoding genetic variants, and Enformer [8] to predict gene expression from DNA sequences. In the following, we discuss various types of molecular data together with a few notable applications of DL without being exhaustive.

### Whole-genome sequences

WGSs are a high-throughput method used to determine an organism’s entire DNA sequence, providing a rich dataset for understanding genetic variation and its potential impact on gene regulation or disease. While regulatory elements such as promoters, transcription factor (TF) binding sites, epigenetic marks, and 3D chromatin interactions (e.g. enhancer–promoter loops) are typically identified through specific experimental assays, computational approaches increasingly aim to predict these features directly from genomic sequence data. Functional genomics experiments produce quantitative signals at each genomic position, measuring protein–DNA binding intensity, chromatin accessibility, or transcription levels that models can learn to predict from DNA sequences alone. The model essentially “sees” the raw DNA sequence as input and learns to output the expected experimental signal, establishing direct sequence-to-function mappings.

Promoter detection is essential for understanding gene transcription, as promoters signal where gene transcription begins [15]. The prediction of TF bindings further enhances our understanding of gene regulation by identifying sequence motifs and the corresponding proteins that bind DNA to modulate gene expression. DL methods, such as DeepTFactor, have proven to be effective for this task and are increasingly used to model TF binding patterns. Gradient-based visualization methods reveal that convolutional layers automatically detect DNA-binding domains and other structural motifs in protein sequences, even without explicit annotation during training [16]. The prediction of epigenetic marks aims at investigating chemical modifications that influence gene accessibility and expression without altering the underlying DNA sequence, playing a key role in processes such as cell differentiation [17]. Similarly, enhancer–promoter interactions contribute to the regulation of complex gene expression programs across tissues and developmental stages. These phenomena can be studied computationally using DL models, such as SPEID, which is specifically designed to predict such interactions [18].

A related task is to decipher the functional consequences of genetic variants, which can occur both within genes and in noncoding regulatory regions. While coding variants are studied more extensively, the majority of the genome is noncoding and plays a key role in gene regulation and expression [19]. Understanding the impact of noncoding variants is particularly challenging, yet essential for uncovering disease mechanisms and informing the development of targeted therapies [20]. Projects like ENCODE [21] and the UK Biobank [22] that include WGS along with other omics datasets provide ideal training datasets to achieve these tasks.

ENCODE systematically maps biochemical events across the genome. Results are presented as parallel tracks aligned with the genomic sequence, showing regions where the vast majority of the human genome participates in at least one biochemical RNA and/or chromatin-associated event in at least one cell type [20]. These comprehensive measurements, including TF occupancy, histone modifications, RNA production, and chromatin accessibility, create natural input–output pairs for machine learning. The models can be trained to predict experimental signals such as ChIP-seq binding peaks or RNA expression levels from DNA sequence, enabling pretext tasks that teach the model relationships within the regulatory code. The UK Biobank provides a complementary resource at population scale, comprising a prospective cohort study with extensive genetic and phenotypic information on $\sim $500 000 individuals, where genome-wide genotype data collected on all participants enable the discovery of genetic associations and investigation of the genetic architecture underlying complex traits [22]. This large-scale genotype–phenotype resource allows models to learn how sequence variants relate to observable characteristics.

When trained on such integrative datasets, SSL can help identify relationships between DNA and function that are not easily discernible through traditional statistical analysis, improving variant prediction accuracies [18].

### RNA sequences

RNA-seq is typically used to measure the relative abundance of RNA molecules within biological samples, providing insights into gene expression, splicing events, and their underlying regulatory mechanisms. RNA-seq data support a wide range of analyses, including differential gene expression, transcript quantification, alternative splicing detection, fusion transcript identification, allele-specific expression, and RNA editing [23].

In this review, we focus on the sequence-level analysis of RNA, with particular emphasis on the prediction and characterization of splicing events. Accurate identification of splice sites is critical for understanding alternative splicing, a process that produces isoforms of RNA transcripts from the same gene. Given that splicing patterns vary across tissues, developmental stages, and diseases, understanding splice site selection is a fundamental step in decoding the complexity of gene regulation. Splice site prediction identifies junctions where noncoding regions (introns) are removed from RNA, producing mature RNA transcripts where only coding sequences (exons) are retained for translation into proteins [24].

Prediction models base their decisions on hierarchical feature extraction from these encoded sequences: convolutional neural networks apply filters that scan the input to identify local motifs, with deeper layers capturing increasingly abstract patterns, from simple dinucleotide signals (GT/AG) to complex regulatory elements like branch points and polypyrimidine tracts, typically located 20–50 nucleotides from splice sites [25]. Predicting branch point sites also allows the identification of intronic points that guide splicing, which is crucial to generate mature RNA transcripts [26]. Cross-species splice site prediction extends this understanding across different organisms, providing insights into conserved splicing mechanisms and evolutionary changes. This principle was leveraged by Spliceator [25], which was trained on multi-species data, teaching the model to recognize evolutionarily conserved sequence features: when models learn from genomic sequences spanning primates to protists, they extract universal splicing signals while maintaining the flexibility to detect organism-specific variations in consensus motifs and regulatory elements. There holds great promise for SSL to be used to pretrain models on large RNA-seq datasets, improving the precision of splicing-related tasks. SSL methods can also improve the analysis of gene expression patterns by identifying underlying structures and relationships within the data, facilitating the discovery of novel regulatory mechanisms.

### Single-cell RNA sequencing

scRNA-seq measures the relative abundance of RNA molecules at the single-cell level, providing information on cellular heterogeneity and function. These data type are crucial for cell clustering and annotation and for improving sequence-based predictions [27]. Identifying different types and states of cells within a heterogeneous population is a key challenge. Computationally, scRNA-seq data present unique characteristics that models must handle: high dimensionality with thousands of genes assayed per cell, and sparse data with often >50% zero values in count matrices [27]. SSL techniques can improve clustering and annotation by learning from large amounts of unlabeled scRNA-seq data. A limitation is that single-cell data often contains high levels of noise and variability. SSL can enhance predictive models by exploiting the intrinsic structure of the data, leading to more robust and accurate predictions.

scRNA-seq is used to perform downstream tasks that explore gene expression and cell identity at the resolution of individual cells, or to investigate heterogeneity within tissues with tasks such as cell pairwise classification, single-cell clustering, and single-cell annotation. Cell pairwise classification involves comparing individual cells to establish relationships, helping to categorize cell types or identify abnormal cells. Single-cell clustering groups cells based on shared molecular characteristics, which is critical for identifying distinct cell populations within complex tissues. Beyond improved clustering accuracy, SSL methods enable critical applications including: automatic cell type annotation by transferring knowledge from reference datasets, discovery of novel cell types through self-supervised similarity learning, and simultaneous denoising through probabilistic modeling [12]. The single-cell annotation assigns biological labels or identities to each group of cells, improving our understanding of tissue composition and function. These techniques are fundamental for advancing knowledge in developmental biology, immunology, and disease pathology [27].

### Protein sequences and protein–protein interactions

Protein sequences are used for a variety of downstream tasks, including protein folding prediction, protein–protein interaction (PPI) prediction, and functional annotation [28]. A landmark example is AlphaFold2, which has demonstrated its efficiency in predicting 3D protein structures directly from sequences [5, 6]. While protein folding has seen major breakthroughs, predicting PPIs from sequence alone remains particularly challenging, as experimental assays to map interactions across the entire proteome are often costly and time-consuming. SSL approaches offer a promising solution by pretraining models on large protein sequence datasets, thereby improving the performance of downstream predictions such as PPI.

SSL models can also help annotate the functions of proteins by learning from vast amounts of PPI data, helping to uncover new biological insights. Other common tasks in protein functional annotation include remote homology, secondary structure prediction, and fluorescence and stability prediction. Remote homology detection methods, which find evolutionarily related proteins with very low sequence similarity, extract features on multiple levels: local sequence composition (k-mers, motifs), evolutionary information from multiple sequence alignments (profiles, PSSM), and alignment-based similarity scores. By integrating these explanatory representations, they can identify distantly related proteins with low sequence identity [29]. For secondary structure prediction, DL models operate on windowed segments of the sequence, using convolutional and recurrent architectures to capture both local structural preferences of individual residues and medium-to-long-range dependencies. Secondary structure prediction identifies elements like alpha-helices and beta-sheets, which form the building blocks of a protein’s 3D structure, crucial for understanding function [30]. Functional traits such as fluorescence prediction and stability prediction assess properties like the protein’s ability to emit light or remain functional under specific conditions [31]. These tasks help to decode protein functions, inform drug design, and improve structural biology insights. In recent years, the life sciences community has accepted DL methods like AlphaFold for tasks such as protein structure prediction and protein discovery [5].

## Self-supervised learning

SSL is a paradigm in DL where the model learns to create supervisory signals from the data itself, eliminating the need for labeled data [32]. This is achieved through pretext tasks, designed to predict some part of the input data from other parts, enabling the model to learn meaningful representations. These tasks exploit the inherent structure in the data and prepare the model for downstream tasks where labeled data are available [9, 10]. SSL allows models to understand the deeper, underlying structure of the data by solving complex pretraining objectives.

SSL has already been successfully applied to other research fields such as computer vision, NLP, speech processing, and robotics. In computer vision, SSL is applied to improve the performance in tasks like image classification, object detection, and segmentation [33]. In NLP, FMs using SSL achieve great results in text classification, language translation, and question answering [34, 35]. In the domain of speech processing, SSL has been applied to speech recognition with WAV2VEC [36] and speaker identification [37]. Finally, SSL has been used in robotics for tasks such as autonomous robot navigation systems [38].

**Figure 1:**
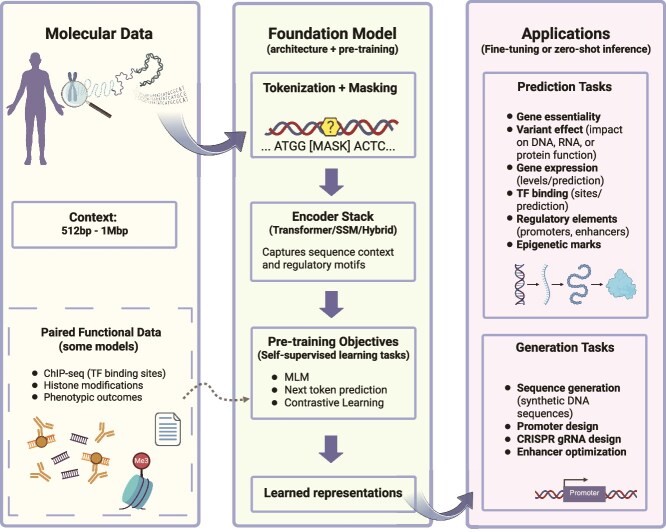
General framework for molecular data FMs and their applications. The architecture consists of three main components: (i) *Genomic Data* input with sequence context ranging from 512 bp to 1 Mbp, optionally supplemented with paired functional data such as ChIP-seq for TF binding sites, histone modifications, and phenotypic outcomes; (ii) *FM* comprising tokenization and masking of DNA sequences, followed by an encoder stack (Transformer, state-space models, or hybrid architectures) that captures long-range dependencies and regulatory motifs through self-supervised pretraining objectives including masked ” (MLM), next token prediction, and contrastive learning, producing learned representations of genomic context; (iii) *Applications* enabling both prediction tasks through fine-tuning or zero-shot inference. Notable examples implementing this framework include DNABERT, nucleotide transformer (NT), HyenaDNA, Evo 2, DNAGPT, and RNABERT. bp, base pair; MLM, masked language modeling.

FMs are large-scale general-purpose neural networks typically built on the transformer architecture [39], which uses a self-attention mechanism that efficiently captures long-range dependencies in the tokenized sequences. Tokenization is the process of converting raw input (such as text or DNA sequences) into discrete units called tokens that a model can process. These tokens may represent characters, subwords, or other meaningful fragments, allowing the model to operate on standardized numerical representations rather than raw data. Transformer-based models have been used extensively in NLP and image processing. FMs are pretrained on extensive datasets with a pretext task using unlabeled data to learn efficient representations and later fine-tuned for multiple downstream tasks where labeled data are available. Figure 1 shows a general framework for an FM using molecular data and its applications.

### Pretraining objectives

#### Sequence-specific pretraining objective

**Figure 2:**
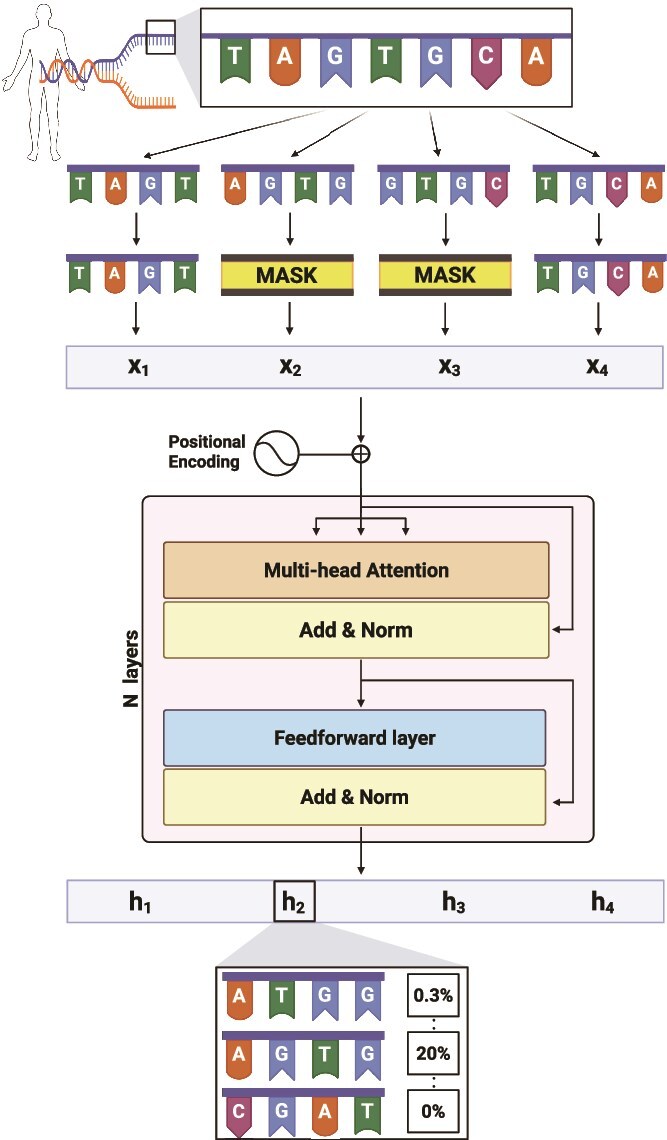
Transformer architecture for genomic sequence analysis using MLM. A DNA sequence is tokenized into individual nucleotides, with random positions masked during training. The input embeddings, combined with positional encoding, are processed through N transformer layers consisting of multi-head attention mechanisms and feedforward networks with residual connections and layer normalization. The model generates contextual representations for each position and predicts nucleotide probabilities at masked positions, enabling SSL of biologically meaningful sequence features. This BERT-like approach allows the model to capture long-range dependencies and contextual information within genomic sequences. x, input embeddings for each token; N, number of transformer layers; h, output embeddings from the transformer.

Sequence data consist of ordered elements modeled as sequences of tokens for DL modeling. This structure naturally enables self-supervised pretraining tasks in which parts of the sequence are masked, corrupted, or otherwise transformed, and the model is trained to either predict the missing elements or remain invariant to such perturbations. BERT introduced MLM as a foundational pretraining objective, in which a subset of tokens is hidden and the model learns to infer them from their context. In MLM, random tokens (nucleotide sequences) within a sequence are replaced by a special mask token, and the model is trained to infer the masked positions from the surrounding left and right context. This objective forces the network to learn the statistical dependencies that govern the structure of the sequences. Because MLM exposes the model to both upstream and downstream context, it supports the acquisition of bidirectional representations that can later be fine-tuned for a wide range of downstream tasks. To better understand this pretext task, Fig. 2 shows a small example of how MLM is applied to a small DNA sequence [9].

A related but distinct SSL pretext task is “next-token prediction” (also called autoregressive or causal language modeling) introduced by GPT. Using next-token prediction the model predicts each token based solely on all preceding tokens in the sequence. Unlike the bidirectional nature of MLM, next-token prediction learns directional, causal dependencies, capturing how elements naturally follow one another in sequence order. This asymmetric learning makes it particularly effective for generative tasks, enabling models to produce novel, coherent sequences rather than simply analyzing existing ones [10].

Training FMs using SSL are potentially highly effective for analyzing DNA sequences, as they can capture long-range dependencies and contextual relationships. Using the same principles used in NLP, the model treats sequences of DNA nucleotides (A, T, C, G) similarly to how it processes sequences of words or tokens in a sentence. These models are potentially useful to represent, e.g. the upstream and downstream of different genes, the different motifs present in a gene, like the start and stop codons, the splice sites, and the coding and noncoding regions of the sequence. All these different elements present in the sequences can be seen as “words” that interact to form “sentences.” FMs could learn an efficient representation of this, as already demonstrated in NLP.

#### Contrastive learning

Contrastive learning is not limited to sequence data and can be applied to virtually any modality, provided that one can construct meaningful positive and negative pairs. However, this often requires a sufficient number of negative examples, which in practice leads to large batch sizes, as seen in methods such as MoCo and SimCLR [11, 12]. Building the pair set is not a trivial task, as the way pairs are defined strongly shapes the representativity and biological fidelity of the final network. At the same time, this step provides an important opportunity to inject domain knowledge, for instance, by constructing biological invariants using the genetic code to input invariant single-nucleotide polymorphisms (SNPs).

**Figure 3:**
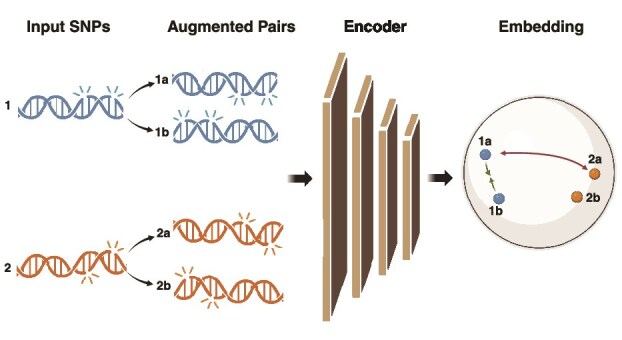
Data augmentation and contrastive learning pipeline for SNPs-based genomic analysis. Input SNP sequences undergo augmentation through random masking and perturbations to generate multiple views of the same genetic variant, creating positive pairs (1a-1b, 2a-2b) that represent the same underlying genomic pattern. These augmented sequences are processed through a shared encoder network to produce low-dimensional embeddings in a latent space. The contrastive learning objective minimizes the distance between embeddings of augmented pairs from the same sample while maximizing separation from different samples, enabling the model to learn robust, invariant representations of genetic variants.

In contrastive learning, the model learns to distinguish between similar (positive), i.e. two views or examples that should be considered similar, and dissimilar (negative) pairs of data points, which represent dissimilar or unrelated examples. The goal is to bring representations of similar data points closer together, while pushing apart representations of dissimilar ones, as shown in Fig. 3 using SNPs as an example. Positive pairs can be constructed through data augmentation (e.g. masking, cropping, shuffling, or noise injection) or by using biologically meaningful relationships, such as different genomic windows from the same regulatory region, alternative views of the same cell in single-cell RNA-seq, or homologous protein sequences. Negative pairs are typically sampled from unrelated sequences, different cells, or nonhomologous proteins, depending on the data type.

Most contrastive methods are pretrained using a contrastive loss such as InfoNCE [40], which encourages the model to maximize the similarity of positive pairs relative to all negatives. From an information-theoretic standpoint, InfoNCE can be interpreted as maximizing a lower bound on the mutual information between representations of the two views, thereby preserving the shared biological signal while filtering out noise or irrelevant variation.

One of the foundational works using contrastive learning is MoCo [11], which is trained using images. MoCo maintains a dynamic dictionary of encoded representations using a momentum encoder. This dictionary allows for a large number of negative samples, making the contrastive task more challenging and effective. Similarly, SimCLR [12] uses data augmentation techniques to create positive pairs and learns representations by maximizing agreement between these augmented views of the same image.

Contrastive learning can be adapted for text data, where sentences, or word embeddings are compared. In the context of DNA sequences, contrastive learning might be applied in the same way as it is applied to text. DNA sequences, like sentences, are ordered strings (composed of nucleotides instead of words), and understanding biological similarity or functional regions can benefit from contrasting different DNA fragments [41].

### Evaluating performance on downstream tasks

After pretraining on pretext tasks, the learned representations are fine-tuned on specific downstream tasks. Commonly, SSL is measured by the performance improvement in these tasks compared with models trained from scratch [42]. Common downstream tasks in NLP include text classification, recognition of named entities, and answering questions [34]. As described in the previous section, for every data type, different common downstream tasks could be used for the evaluation performance of the different SSL models.

By understanding and applying these SSL paradigms, researchers can unlock new insights from the vast amounts of molecular data, enhancing our understanding of complex biological processes and improving the accuracy of omics analyses.

## Survey methodology

In this review, a comprehensive survey of the literature is performed on SSL and its applications using molecular data. It starts with an extensive search of peer-reviewed journals, conference papers, and preprints from PubMed, arXiv, bioRxiv, and Google Scholar. Keywords used in the search included “SSL,” “molecular data,” “genomics,” “DNA sequence analysis,” “contrastive learning,” “transformed–based models,” “transformers in genomics,” “transformers and molecular data,” “FMs in genomics,” “FMs and molecular data,” “SSL in biology,” and “DL and molecular data.” The search was not restricted by publication date to capture both foundational and recent advancements in the field.

Papers were selected based on their relevance to SSL methodologies and their applications in omics. Inclusion criteria included empirical studies, and theoretical papers that provide significant insights or results related to the application of SSL in omics data analysis. Studies that did not explicitly apply SSL to omics or lacked sufficient methodological details were excluded. Selected papers were categorized into different sections based on the type of molecular data used (e.g. DNA sequences, RNA sequences, and single-cell, proteins).

The extracted data were analyzed to identify common trends, unique approaches, and the overall impact of SSL on molecular data analysis. The main findings were synthesized into a cohesive narrative, emphasizing the contributions and potential of SSL in advancing omics research. For each selected study, we extracted key information including the type of SSL approach used, tokenization strategies, pretext tasks, downstream tasks, datasets, hardware requirements, and training times. We also noted any publicly available repositories in GitHub and HuggingFace to facilitate reproducibility and further research.

To ensure the accuracy and relevance of the review, we cross-referenced our findings with expert opinions in DL and omics. This systematic approach to analyzing the literature allowed us to present a detailed and structured review of SSL using molecular data, providing valuable insights for researchers and clinicians.

## Applications of self-supervised learning using molecular data

SSL has shown immense potential in improving the analysis of molecular data by leveraging large-scale, unlabeled datasets to learn meaningful representations. These representations can then be fine-tuned for specific downstream tasks. This section describes how SSL has been applied to different types of molecular data, including WGS, RNA-seq, scRNA-seq, and protein sequences. The main characteristics of all the models presented are summarized in Tables 1 and 2.

**Table 1:** Summary of the models using SSL with different types of molecular data, presenting the datasets and pretext tasks used for pretraining alongside the downstream tasks used to analyze model performance.

Model	Pretext task	Downstream task	Pretraining dataset
DNABERT (86M–89M) [43]	MLM	Predicting promoter regions, TF binding sites, recognizing canonical and noncanonical splice sites, identify functional genetic variants	The human reference genome GRCh38, nonoverlap splitting, random sampling, sequence lengths 10–510, 2.75B nucleotide bases
DNABERT-2 (117M) [44]	MLM	Promoter detection, species classification, TF, splice site, epigenetic marks prediction, enhancer promoter interaction	DNABERT human genome dataset, the multi-species genome dataset: 135 species, 6 categories, 32.49B nucleotide bases
DNABERT-S [45]	MLM and, curriculum contrastive learning	Species clustering, classification	Reference genomes from GenBank: 47 923 pairs 17 636 viral, 1 million pairs 5011 fungi, 1 million pairs 6402 bacteria, DNA sequences 10 000 bp
NT (480M–2537M) [46]	MLM	Epigenetic marks prediction, promoter sequence prediction, enhancer sequence prediction, splice site prediction	Human reference genome GRCh38: 3.2 billion nucleotides 1000G dataset: 20.5 trillion nucleotides, multispecies dataset: 850 species, 174 billion nucleotides,
AgroNT (1B) [47]	MLM	Polyadenylation site prediction, Splicing site, chromatin profiles prediction, noncoding RNA, promoter strength prediction, tissue gene expression level	Plant reference genomes, Ensembl Plants database 10.5 million genomic sequences across 48 different species
SegmentNT [48]	MLM	Finetuning NT for segmentation of DNA sequences at nucleotide resolution	Annotations at nucleotide-level, 14 types of genomic elements, the human genome, 20.48M sequences
HyenaDNA (0.4–6.6 M) [49]	Next-token prediction	Single nucleotide resolution, chromatin profile prediction, BiotypeEmbeddings, species classification	The human reference genome
Evo 2 (7B–40B) [50]	Next-token prediction,	Variant effect scoring and prediction, Exon/intron classification Gene and lncRNA essentiality	OpenGenome2 dataset: 2.4T tokens, (7B), 9.3T tokens, (40B) multi-species genomic sequences
DNAGPT (0.1B–3B) [51]	Next-token prediction GC content prediction sequence order prediction	GSR recognition (PAS/TIS), mRNA expression prediction, promoter prediction	All mammalian genomes GenBank; 200B bases
Self-GenomeNet [52]	Contrastive learning	Effector gene prediction, bacteriophage, protozoa-fungui prediction, classify prokaryotic and eukaryotic viruses, identify effector proteins	Bacterial dataset containing bacterial genomes GenBank22 and RefSeq23, 83 billion nucleotides
SNP2VEC [53]	MLM	Alzheimer’s disease prediction	The human reference genome GRCh37 dbSNP version 153
RNABERT [54]	MLM and structural alignment learning (SAL)	RNA structural alignment, RNA family clustering	76 237 human-derived small ncRNAs Rfam, BRAliBase2.1 k2
SpliceBERT [55]	MLM	Splice site prediction, predicting BP sites, zero-shot variant effects prediction, cross-species splice site prediction	RNA sequences 72 vertebrates Reference genomes, gene annotations UCSC, >2 million sequences, $\sim $65 billion nucleotides
scAnCluster [56]	Cell pairwise classification task	Single-cell clustering, single-cell annotation	2 groups of datasets from pancreas tissue and retinal tissue, a total of 107 211 cells
contrastive-sc [57]	Contrastive learning	Cell clustering, KMeans1 and Leiden2 community detection	24 Simulated dataset 15 real-world scRNA-seq datasets from scziDesk and scDeepCluster
CPCProt [58]	Contrastive learning	Remote homology, secondary structure, fluorescence, stability	Protein domain sequences Pfam database. 32 207 059 Pfam amino acid sequences
SMG [59]	SMG reconstruction	Cancer gene identification, identification of CGs, essential genes, healthy driver genes, identification disease subnetworks across PPI networks	29 446 samples covering 16 cancer types TCGA datasets PPI Networks from multiple public databases CPDB, STRING-db, IRefIndex, Multinet, PCNet

MLM, masked language modeling; GSR, genomic signals and regions; PAS, recognition of polyadenylation signals; TIS, translation initiation sites; CG, cancer genes; PPI, protein–protein interaction; TCGA, the Cancer Genome Atlas program; GC, guanine–cytosine.

**Table 2:** Summary of the models using SSL with different molecular data, providing a comprehensive overview of the data types, pretraining data preparation, hardware, training time, and useful model repositories.

Model	Data type	Data preparation for pretraining	Hardware and training time	Repositories
DNABERT (86M–89M) [43]	DNA sequences	Overlapping 3-mers, 4-mers, 5-mers, and 6-mers tokenization	8 Nvidia 2080Ti GPUs –	github.com/ jerryji1993/DNABERT huggingface.co/ zhihan1996
DNABERT-2 (117M) [44]	DNA sequences	BPE, variable length tokens	8 NVIDIA RTX 2080Ti V.S GPUs 14 days	github.com/MAGICS–LAB/ DNABERT_2 huggingface.co/zhihan1996/ DNABERT–2–117 M
DNABERT-S [45]	DNA sequences	BPE, variable length tokens	8 NVIDIA A100 80GB GPUs 48 h	github.com/ MAGICS–LAB/DNABERT_S huggingface.co/ zhihan1996/DNABERT–S
NT (480M–2537M) [46]	DNA sequences	6-mers tokenization	Cambridge-1 Nvidia 500M: 8 A100-1 day 2.5B: 128 A100-28 days	github.com/instadeepai/ nucleotide-transformer huggingface.co/ InstaDeepAI
AgroNT (1B) [47]	DNA sequences	Nonoverlapping 6-mer tokenization, the model takes as input a sequence of 1025 tokens	TPU v4-1024 machine 512 devices -	github.com/instadeepai/ nucleotide–transformer huggingface.co/InstaDeepAI/ agro–nucleotide–transformer–1b
SegmentNT [48]	DNA sequences	6-mers tokenization	8 GPU H100 >20 h	github.com/instadeepai/ nucleotide–transformer huggingface.co/ InstaDeepAI/segment_nt
HyenaDNA (0.4–6.6 M) [49]	DNA sequences	Each nucleotide is a token and “N” (nonspecific nucleotide)	8 Nvidia A100 (80 GB) GPUs	github.com/ HazyResearch/hyena–dna huggingface.co/LongSafari
Evo2 (7B–40B) [50]	DNA sequences	Each nucleotide is a token	Nvidia H100 Tensor Core GPU	github.com/arcinstitute/evo2 huggingface.co/datasets/arcinstitute/opengenome2
DNAGPT (0.1B–3B) [51]	DNA sequences	Nonoverlapping k-mers Numerical Token Integration	8$\times $V100 (0.1B) 16 V100 (3B)	github.com/ TencentAILabHealthcare/DNAGPT
Self-Geno- meNet [52]	DNA sequences	Reverse-Complement (RC) 150 nt sequences: patch size 24, stride 6 1000 nt sequences: patch size 40 stride 20	GeForce RTX 2080 Ti GPU	github.com/GenomeNet/ Self–GenomeNet
SNP2VEC [53]	DNA sequences diploid	Transformer variant with linear-attention mechanism and BPE tokenization Tokens represented as “X1/X2” (diploid)	5 2080Ti GPUs Intel(R) Xeon(R) Silver 4210 CPU	github.com/HLTCHKUST/ snp2vec
RNABERT [54]	RNA sequences	Single-nucleotide tokenization BERT masking Sequence pairs for SAL	GPU: Tesla v100 CPU: Intel(R) Xeon(R) Gold 6148	github.com/ mana438/RNABERT/
SpliceBERT [55]	RNA sequences	Each nucleotide (A, G, C, T/U) as a token	First stage: 8 Nvidia V100 GPUs—7 days second stage: 4 Nvidia V100 GPUs—3 days	github.com/ chenkenbio/SpliceBERT
scAnCluster [56]	Single-cell RNA sequences	Merge source dataset–target dataset by shared gene names Top 1000 variable genes Transform data into z-score data	–	github.com/ xuebaliang/scAnCluster
contrastive-sc [57]	Single-cell RNA sequences	scziDesk preprocessing using package Scanpy Top 500 most variable genes	GeForce RTX 2060 GPU	github.com/ ciortanmadalina/ contrastive–sc
CPCProt [58]	Protein sequences	Protein sequences are divided into patches of size 11	single GPU	github.com/ amyxlu/CPCProt
SMG [59]	Proteins, gene: PPI graph	Each gene as a graph node Construct edges between nodes by PPI Networks transformed into graph data	Nvidia A100 GPU	github.com/C0nc/SMG

BPE, byte pair encoding; SAL, structural alignment learning; PPI, protein–protein interaction; GPU, graphics processing unit; CPU, central processing unit.

### Whole-genome sequences

WGS provides comprehensive information about the entire genetic makeup of an organism. Self-supervised methods have been employed to extract meaningful representations from these vast datasets, aiding in various genomic analyses.

The earliest genomic models using SSL adopted k-mer tokenization strategies borrowed from traditional bioinformatics. DNABERT [43] pioneered the adaptation of the BERT model to DNA sequences by pretraining on large-scale genomic datasets using an MLM as pretraining objective. The MLM objective forces the model to predict masked k-mers by learning the statistical dependencies, the “grammar” of genomic sequences, inherently capturing patterns such as TF binding motifs, splice sites, and codon structures. DNABERT represents sequences as overlapping k-mers (typically $k=6$, though $k=3$ showed similar performance), creating a vocabulary of all possible k-mer permutations plus special tokens ([CLS], [PAD], [UNK], [SEP], [MASK]), resulting in $4^{k}$ + 5 = 4101 total vocabulary tokens, reaching a context window of 1k nucleotides. Building on this foundation, NT [46] employed the same BERT methodology with 6-mer tokenization and MLM objective pretrained on diverse datasets, but with a context window of 12k nucleotides. NT demonstrated that transformer models yield transferable, context-specific representations of nucleotide sequences that enable accurate molecular phenotype prediction even in low-data settings. Within the NT framework, specialized variants were developed: AgroNT [47] focused on plant genomics by pretraining on 48 crop species genomes, while SegmentNT [48] retrained NT parameters to predict the location of 14 different genomic element types at single-nucleotide resolution, enhancing granular prediction capabilities at the nucleotide level. SNP2Vec [53] is also inspired by BERT and uses MLM as an objective task. SNP2Vec leverages SNP data to learn representations of genomic variations using SSL, which is crucial for genome-wide association study (GWAS). SNP2VEC leverages genomic variations from diploid sequences during pretraining. The model demonstrates its effectiveness in predicting Alzheimer’s disease risk.

In parallel, instead of using transformer-based models, Self-GenomeNet [52] is an SSL method that uses a contrastive learning pretext task tailored for the unique characteristics of DNA sequences. The key innovation is that Self-GenomeNet exploits the reverse complement (RC) property of DNA, recognizing that both a sequence and its RC are biologically meaningful. During pretraining, the model takes a segment of a sequence and is trained to predict the RC of another segment within the same sequence. This forms a contrastive task where the model distinguishes true pairs (input and correct RC) from false pairs, greatly enhancing the representations learned from unlabeled data. This enables a symmetric design of the SSL method, reducing the number of model parameters and lowering the risk of overfitting. Additionally, the model enhances the learned representations by incorporating RC awareness by predicting RC targets.

The limitations of k-mer tokenization, which are computational inefficiency, information leakage from overlapping k-mers during masking, and vocabulary explosion with increasing k, motivated a paradigm shift toward more flexible tokenization schemes. DNAGPT [51] introduced byte-level tokenization for DNA sequences, which led to a bigger vocabulary of 8k words, but enabled a bigger context window of 25k nucleotides during the large pretraining phase. Byte pair encoding (BPE) emerged as a data-driven tokenization approach that iteratively merges the most frequent pairs of characters to build an optimal vocabulary, offering three different advantages. First, flexible vocabulary size that adapts to sequence statistics, second, efficient representation of common genomic motifs as single tokens, and third, reduced sequence length compared to single-nucleotide tokenization while avoiding k-mer overlap issues. DNAGPT employs a hierarchical token structure with sequence tokens (DNA sequences), number tokens (numerical values), instruction tokens (task prompts like “Human” or “Bovine”), classification tokens (“A” for True, “N” for False), and connection tokens (“+” for aggregation, “=” for input–output relations). In practice, this allows the model to take DNA sequences along with additional numerical or experimental inputs, such as mRNA half-life, and task instructions, and then predict relevant outputs like mRNA expression levels. Moreover, they incorporated multi-task pretraining, including next-token prediction, RC detection (key innovation of Self-GenomeNet [52]), and guanine–cytosine (GC) ratio regression. This enabled cross-species adaptability for tasks like polyadenylation signal recognition and mRNA expression prediction. DNABERT-2 [44] employed BPE tokenization, justifying it as an improvement due to avoiding data leakage during masking (since tokens don’t overlap as they used to for k-mers), avoiding the catastrophic embedding shifts caused by single-nucleotide insertions (which change all downstream k-mers, hence mapping them to completely different vocabulary entries), and reducing sequence lengths by $\sim $5-times compared with k-mers tokenization. DNABERT-2 introduced the genome nderstanding evaluation (GUE) benchmark for multi-species genomes, highlighting the scalability of SSL approaches. Ablation studies demonstrated that pretraining substantially improves performance, with metrics increasing by $\sim $20% on average across downstream tasks compared to nonpretrained versions. Building on DNABERT-2, DNABERT-S [45] demonstrates the applicability of curriculum contrastive learning (C2LR) to tasks such as species classification and clustering. In C2LR, the model is first trained on easy sequence pairs and then progressively on harder, more ambiguous pairs, enabling it to learn discriminative features and generalize better across DNA sequences.

The most recent generation of models has achieved single-nucleotide resolution at unprecedented context lengths by abandoning traditional transformer attention mechanisms in favor of hybrid architectures. HyenaDNA [49] processes DNA sequences $\sim $1 million tokens at single-nucleotide resolution, overcoming the quadratic scaling limitations of attention mechanisms. HyenaDNA introduces the Hyena operator, which replaces self-attention with implicit long convolutions combined with data-controlled gating, achieving sub-quadratic time complexity through fast convolution algorithms (e.g. FFT). Trained using a length curriculum (progressively increasing sequence length) and gradient checkpointing for memory efficiency, and pretrained solely on the reference human genome, HyenaDNA demonstrates the first use of in-context learning in genomics, enabling adaptation to new tasks without updating pretrained weights, but rather injecting into the input sequence learnable tokens that will be used for the downstream analysis. Evo2 [50], further advances this paradigm by integrating hybrid convolutions (with short, medium, and long receptive fields) with sparse attention blocks inserted at intervals and gating mechanisms for dynamic mixing. Evo2 uses single-nucleotide, byte-level tokenization (A, C, G, T, N) with special stitching tokens to combine fragmented sequences during mid-training. A critical innovation is the two-stage training curriculum: pretraining on shorter contexts (1k–8k tokens) around transcription start sites (TSS) on trillions of tokens, followed by mid-training on long-context genome modeling $\sim $1M tokens. Evo2 employs reweighted loss (0.1 for repetitive versus 1.0 for nonrepetitive regions) to reduce the dominance of repetitive genomic regions, and uses multi-stage rotary position embedding (RoPE) scaling with higher frequencies during the mid-phase to preserve stability at million-token contexts. Pretrained on the OpenGenome2 dataset, which was released by the authors of Evo2, the model effectively captures long-range dependencies, enabling accurate zero-shot variant effect prediction and the first coherent genomic sequence generation. The OpenGenome2 is a new curated, nonredundant nucleotide dataset containing over 8.8 trillion nucleotides from bacteria, archaea, eukaryotes, and bacteriophages publicly available.

### RNA sequences

RNA sequencing (RNA-seq) data provide information on the abundance and diversity of RNA molecules. SSL techniques have been used to characterize the diversity of RNA transcripts, particularly in predicting RNA splicing.

RNABERT [54] introduces a BERT-style base embedding specifically targeted to RNA structural tasks. It explores the idea of alternating as pretraining objectives, MLM, and structural alignment learning (SAL). This hybrid pretraining produces position-aware per-base embeddings that capture not only nucleotide identity but also local secondary-structure and context signals. This approach, termed “informative base embedding,” has shown effectiveness in the RNA structural alignment and RNA family clustering downstream tasks. When combined with a simple Needleman–Wunsch alignment [60] on the learned embeddings, RNABERT achieves structural alignment with a time complexity of O(n2) instead of the O(n6) in comparison with the classical Sankoff-style algorithms.

Building on RNABERT, SpliceBERT [55] employs a BERT architecture pretrained with MLM on 2 million primary RNA sequences from 72 vertebrate species, encoding each nucleotide as a token to learn context-dependent, evolution-aware representations. SSL enables the model’s hidden states and attention patterns to capture conserved sequence features around splice sites and branchpoints, providing biologically meaningful embeddings for downstream tasks. As a result, SpliceBERT supports zero-shot prediction of variant effects on splicing, improves branchpoint detection in humans, and achieves strong cross-species splice site prediction, outperforming models trained on single-species data and highlighting the value of multi-species genomic language modeling.

### Single-cell RNA sequencing

Single-cell technologies capture the heterogeneity within biological samples at a high resolution, providing insights into cellular diversity and function. SSL methods have been applied to single-cell data to improve the accuracy of cell type identification and the reconstruction of developmental pathways.

scAnCluster [56] combines supervised, SSL, and unsupervised learning to jointly perform cell clustering and cell-type annotation on scRNA-seq data. It explicitly uses labeled reference datasets, but is first pretrained on both reference and unlabeled query cells through a pairwise similarity task, where the model predicts whether two cells are similar or dissimilar without needing labels, providing a label-efficient self-supervised signal. After this pretraining, scAnCluster uses the reference labels to guide annotation while still allowing the discovery of novel cell types absent from the reference—a key advantage when analyzing new tissues or conditions.

Extending on the idea of pretraining with similarity constraints, contrastive-sc [57] uses contrastive learning as a pretext task for scRNA-seq clustering. For each cell, two augmented views are constructed by randomly masking different subsets of genes, and the model is trained to bring representations of the two views of the same cell closer while pushing apart representations of different cells, yielding robust, noise-tolerant embeddings. In systematic comparisons against 11 state-of-the-art scRNA-seq clustering methods on both simulated and real datasets, contrastive-sc achieves competitive clustering performance while remaining computationally efficient and scalable to large single-cell datasets.

### Protein sequences and protein–protein interactions

Protein sequences determine protein function, structure, but also the molecular interaction partners. SSL approaches have been applied to predict protein function and PPI, contributing to the understanding of regulatory networks within cells.

CPCProt [58] is an SSL contrastive learning method developed to generate efficient and informative protein sequence embeddings. Instead of relying on large-scale multiple sequence alignments or traditional supervised labels, CPCProt leverages mutual information maximization through a contrastive loss: the model is trained to predict whether local patches of a sequence logically and sequentially follow a given global context, mimicking how biological motifs and domains are organized within proteins. The approach involves segmenting a protein sequence into patches and using an autoregressive encoder to capture the context up to a certain point. For pretraining, CPCProt distinguishes true patches (those that occur in the same context in the actual protein) from negative samples drawn randomly from the dataset, forcing the model to learn meaningful representations that encode both local and global sequence dependencies.

Building on the success of CPCProt for protein sequences, recent advances have extended SSL to structured biological data such as PPI networks. The self-supervised masked graph (SMG) learning approach [59] is a novel DL framework designed for cancer gene identification from multi-omics PPI networks. In the pretext training phase, nodes in the multi-omic PPI graphs are randomly replaced with a special mask token at a 50% masking ratio. A graph neural network (GNN) autoencoder is then trained to reconstruct the masked nodes by leveraging information from their neighboring nodes, effectively capturing the complex interaction relationships and preserving topological graph structure in a self-supervised manner. Following pretraining, the learned GNN encoder is fine-tuned with task-specific layers on several downstream biological prediction tasks: identifying cancer genes, essential genes, healthy driver genes, and disease subnetworks, spanning multiple independent PPI networks.

## Discussion

The application of SSL to molecular data has shown significant promise in overcoming the challenges associated with analyzing large-scale and complex datasets. Using vast amounts of unlabeled molecular data, SSL methods can learn informative representations that capture the underlying biological patterns and regulatory elements. This review highlights the effectiveness of different SSL pretraining objectives, such as MLM, next-token prediction, and contrastive learning, in extracting meaningful features from molecular data.

When comparing SSL methods applied to different types of omics data, evaluating and comparing their performance is challenging. These models vary widely in parameter scales ($\sim $1B to >40B), pretraining datasets (single-genome to multi-species), fine-tuning strategies, and context-window lengths (1k to 1M nucleotides), optimizing each model for distinct task granularities and complicating direct head-to-head benchmarking. Similar issues have been observed in computer science and biomedical fields, where differences in preprocessing, evaluation metrics, and model sizes often obscure true model-to-model comparisons [61]. These inconsistencies also make reproducing published results difficult, as small deviations in pipelines, tokenization, training objectives, or hyperparameters can substantially affect downstream performance. Well-established benchmarks such as SuperGLUE [62], ImageNet [63], CAFA [64], and DREAM [65] highlight the value of standardized datasets and evaluation protocols in ensuring fair, reproducible comparisons.

DNABERT-2 introduces GUE, a dataset for benchmarking [44]; however, it comprises only very specific tasks using only DNA data. Therefore, the primary difficulty lies in the lack of recognized standardized benchmarking tools and datasets across these diverse methodologies. Unlike fields like NLP or image recognition, where well-established benchmarks exist, the genomics and omics fields are still developing appropriate domain-specific evaluation frameworks. Consequently, evaluating the effectiveness of these models often relies on custom datasets or specific tasks, which limits comparability across studies and makes it harder to determine which model performs better in a broad biological context. Therefore, creating unified benchmarks is essential for advancing SSL in omics research, as it would allow omics models to be tested and evaluated in an unbiased way through organized challenges and contributions from independent researchers [66].

Across molecular data modalities, FMs’ pretraining objectives, such as MLM and next-token prediction, and contrastive learning, provide two distinct pathways for representation learning that emphasize different biological signals. FMs pretext tasks encourage models to learn the internal structure of biological sequences or profiles by predicting missing elements from the surrounding context; as a result, they capture fine-grained local dependencies, such as motifs in DNA, residue patterns in proteins, or structural constraints in RNA [9]. In contrast, contrastive learning focuses on the relationships between samples by pulling together different views or perturbations of the same biological entity while pushing apart unrelated ones. This makes it particularly effective for domains where the biologically meaningful signal lies in similarity patterns across samples or cells, rather than in local token-level composition [41]. In practice, MLM and next-token prediction excel when the goal is to model the intrinsic syntax of a sequence [46]. On the other hand, contrastive learning is advantageous when the aim is to learn robust and invariant representations of higher-level biological states [52].

Despite the described advancements, there are still challenges and opportunities for further research. In particular, future work can benefit from more biologically informed and task-relevant pretext tasks. For instance, predicting 3D chromatin contact profiles (e.g. Hi-C or Micro-C submatrices) from 1D genomic sequence to force the model to capture long-range enhancer–promoter interactions, performing contrastive learning on experimentally supported enhancer–promoter pairs versus distance-matched negatives or learning cross-modal prediction tasks such as estimating gene expression levels from the combination of sequence and chromatin accessibility [18, 67]. Integrating multi-omics data and exploring advanced architectures can further enhance the capabilities of SSL in omics. Integrating data from multiple omics layers, such as genomics, transcriptomics, and epigenomics [68] using DL techniques can provide a more comprehensive understanding of biological systems and disease mechanisms. SSL can facilitate this integration by learning comprehensive representations from combined datasets. By pretraining models on tasks that involve multiple data types (e.g. predicting gene expression from both DNA and epigenetic data), SSL can improve the integration and analysis of multi-omics datasets using multi-modal learning techniques, as already demonstrated using the SSL feature embeddings from different sources of data like images and text [69–71].

Developing DL models that offer greater interpretability and transparency could help researchers and clinicians understand the biological relevance of their findings and make more informed decisions. Practical strategies could include analyzing attention patterns to identify whether models focus on regulatory elements; applying *in-silico* mutagenesis or gradient-based attribution methods to determine how specific nucleotide changes alter model predictions; performing motif discovery on highly influential regions to link predictions to known TF binding events. Additional approaches, such as probing classifiers for regulatory features or saliency analysis on predicted 3D contacts can further provide insight into how models encode biological information. The continued development of more accurate and robust predictive models will improve the identification of genetic risk factors and the prediction of disease outcomes, leading to better prevention and management strategies.

The application of SSL using molecular data is still in its early stages, but the potential is very high. Future research can explore more sophisticated pretraining objectives and larger and more diverse omics datasets. Additionally, integrating SSL with other learning paradigms, such as active learning and federated learning, can further enhance the analysis and interpretation of molecular data. Following the advancements that SSL provided to NLP, computer vision, etc., SSL now holds great potential to unlock new insights from the vast amounts of molecular sequence data, advancing our understanding of complex biological processes and improving the accuracy of omics analyses. The ongoing development and application of SSL methods hold great promise for the future of omics and personalized medicine.

## Conclusion

SSL has emerged as a powerful tool for analyzing molecular data, offering significant improvements in understanding and interpreting complex biological systems. By leveraging large-scale unlabeled datasets, SSL methods can learn meaningful representations that enhance various omics analyses, predicting the impact of genetic variants, splicing, protein function to cell type annotation based on single-cell RNA-seq. This review has highlighted the potential of SSL using molecular data, showcasing 17 key studies and their contributions to the field by applying FMs, with MLM and next-token prediction as pretraining objective and contrastive learning.

The continued development and application of SSL methods hold great promise for advancing omics research and personalized medicine. By addressing the challenges associated with analyzing vast and complex molecular data datasets, SSL can unlock new insights into genetic regulation, disease mechanisms, and evolutionary processes. Future research should focus on developing doman-specific pretraining objectives tailored for the high-dimensionality and complexity of omics data, integrating multi-omics data, and exploring advanced architectures to further enhance the capabilities of SSL using molecular data.

**Key points**

- Provides the first comprehensive survey of self-supervised learning (SSL) methods applied to molecular sequence data, addressing a gap despite rapidly growing interest in the field,
- Reviews foundational SSL principles and model architectures, including foundation models, and explains their relevance for omics research,
- Categorizes SSL applications across major omics data types, whole-genome sequencing, RNA sequencing, and single-cell RNA sequencing, and protein sequences, summarizing pretraining objectives, downstream tasks, datasets, and available code repositories to support reproducibility.

## Data Availability

No new data were generated or analysed in support of this research.

## Use of large language models

We declare the use of large language models as:

- an aid to correct written text (spell checkers, grammar checkers),
- an aid to language translation.
